# Cellular Carcinogenesis: Role of Polarized Macrophages in Cancer Initiation

**DOI:** 10.3390/cancers14112811

**Published:** 2022-06-06

**Authors:** Ram Babu Undi, Adrian Filiberti, Naushad Ali, Mark M. Huycke

**Affiliations:** 1 Department of Radiation Oncology, University of Oklahoma Health Sciences Center, Oklahoma City, OK 73104, USA; rambabu-undi@ouhsc.edu (R.B.U.); adrian-filiberti@ouhsc.edu (A.F.); 2 Stephenson Cancer Center, University of Oklahoma Health Sciences Center, Oklahoma City, OK 73104, USA; naushad-ali@ouhsc.edu; 3 Department of Internal Medicine, Section of Digestive Diseases and Nutrition, University of Oklahoma Health Sciences Center, Oklahoma City, OK 73104, USA

**Keywords:** carcinogenesis, macrophage activation, parainflammation, inflammaging, DNA damage, mutation, macrophage modulation

## Abstract

**Simple Summary:**

Inflammation is a hallmark of many cancers. Macrophages are key participants in innate immunity and important drivers of inflammation. When chronically polarized beyond normal homeostatic responses to infection, injury, or aging, macrophages can express several pro-carcinogenic phenotypes. In this review, evidence supporting polarized macrophages as endogenous sources of carcinogenesis is discussed. In addition, the depletion or modulation of macrophages by small molecule inhibitors and probiotics are reviewed as emerging strategies in cancer prevention.

**Abstract:**

Inflammation is an essential hallmark of cancer. Macrophages are key innate immune effector cells in chronic inflammation, parainflammation, and inflammaging. Parainflammation is a form of subclinical inflammation associated with a persistent DNA damage response. Inflammaging represents low-grade inflammation due to the dysregulation of innate and adaptive immune responses that occur with aging. Whether induced by infection, injury, or aging, immune dysregulation and chronic macrophage polarization contributes to cancer initiation through the production of proinflammatory chemokines/cytokines and genotoxins and by modulating immune surveillance. This review presents pre-clinical and clinical evidence for polarized macrophages as endogenous cellular carcinogens in the context of chronic inflammation, parainflammation, and inflammaging. Emerging strategies for cancer prevention, including small molecule inhibitors and probiotic approaches, that target macrophage function and phenotype are also discussed.

## 1. Introduction

The majority of cancers develop in a multi-stage fashion defined by initiation, promotion, progression, and metastasis. Within each stage, the transition from healthy somatic cells to metastatic malignancy is regulated in part by host immunity. Inflammation, as driven by host immunity, is considered an essential hallmark of cancer [1]. For many cancers, inflammation is an enabling characteristic that precedes malignant transformation with a subsequent shift to immunosuppressive tumor microenvironments that promote survival, growth, and metastasis. In this review, we focus on the role of chronic inflammation, inflammaging, and parainflammation in cancer initiation, with an emphasis on macrophages as key cellular drivers during the earliest stages of carcinogenesis [2]. The role of tumor-associated macrophages in the maintenance and growth of extant cancers, however, is not considered. We use the term cancer initiation to refer to cells with acquired mutations in genomic DNA, altered phenotypes, and proliferative potential [3]. Despite recent progress, the role of immune cells in cancer initiation is not fully defined [4]. This review discusses the emerging role of chronically polarized macrophages in cancer initiation. Chronic polarization refers to cells with phenotypes that persist well beyond normal homeostatic responses to infection or injury. Finally, agents that target macrophages are considered as newer approaches to cancer prevention.

## 2. Macrophages in Innate Immunity

The innate immune system is the body’s first line of defense and helps identify and eliminate “non-self” substances such as invading pathogens. The primary cellular components of the innate immune system are monocytes, macrophages, basophils, dendritic cells, eosinophils, mast cells, neutrophils, and natural killer cells. Precursors to these components arise from bone marrow stem cells via granulocyte-monocyte maturation and are found in the circulation as monocytes [5,6]. These cells differentiate into macrophages upon entering tissue compartments. Monocytes and macrophages are distinguished by surface markers such as CD11b, CD68, F4/80, CD163, CD206, and Ly6C, among others [7]. In contrast to bone marrow derived-macrophages, tissue-resident macrophages largely originate in the yolk sac, express many niche-specific functions, and have long lifespans [8,9].

Macrophages possess a high degree of plasticity that allow for adaptation to local environmental cues [10,11]. Switching to specialized functions in response to specific external signals is termed polarization. Macrophage plasticity and polarization allow for a finely tuned check-and-balance between progression and inhibition of inflammatory responses. Under homeostatic conditions, intestinal and liver macrophages are profoundly anergic despite retaining robust phagocytic and bactericidal activity [12,13]. The maintenance of an “M0” or “non-polarized” phenotype is largely due to interleukin (IL)-10 and transforming growth factor (TGF)-β. Tolerogenic cells can be polarized to a pro-inflammatory M1 phenotype by a variety of signals, including pathogens, antigens (e.g., lipopolysaccharide [LPS]), and cytokines (e.g., interferon [INF]-γ, tumor necrosis factor [TNF]-α, and granulocyte-macrophage colony-stimulating factor 2 [CSF2]). However, abnormal, excessive, or chronic M1 polarization can lead to tissue damage and chronic inflammation [14,15]. 

M1 polarized macrophages coordinate inflammatory responses through many potent cytokines (e.g., TNF-α, IL-1α, IL-1β, IL-12, IL-18, and IL-23) [16]. The cytokine IL-6, as expressed through nuclear factor (NF)-κB/Stat3 signaling, facilitates cancer initiation by enhancing the proliferation of tumor-initiating cells [17]. Conversely, Toll-like receptor (TLR)-induced cytokine pathways are broadly inhibited when macrophages express growth arrest-specific gene 6 (GAS6), a soluble ligand for Tyro3-Axl-Mer receptors kinase [18]. Knockout of *Gas6* in *Apc*^Min/+^ mice increased macrophage infiltration in the intestinal tract, elevated cytokine levels, induced cyclooxygenase (COX)-2, and enhanced tumor initiation [19].

In contrast, M2-polarized macrophages display anti-inflammatory and immunosuppressive functions. The phenotype is induced by IL-4, IL-10, IL-13, IL-21, IL-33, TGF-β, glucocorticoids, and macrophage colony-stimulating factor (CSF1). M2 macrophages are involved in tissue regeneration, wound healing, and parasite clearance. These cells produce polyamines and secrete high levels of IL-10, prostaglandin (PG) E_2_, and TGF-β. M2 macrophages can be subdivided into several different phenotypes (e.g., M2a, M2b, M2c, and M2d), depending on specific polarizing signals (for reviews see [20,21]).

## 3. Macrophages in Tissue Homeostasis

Macrophages maintain homeostasis in response to pathogens, tissue injury, and dying cells and provide negative feedback to prevent chronic inflammation. Resolution of inflammation requires the restriction of pro-inflammatory mediators, reduction in immune cells at sites of inflammation, and restoration of tissue integrity [22]. Resolving tissue injury reduces the risk of malignancy associated with chronic inflammation. Homeostatic or M0 macrophages help prevent inflammation by engulfing dead cells, tissue debris, and/or bone through the process of efferocytosis [23,24]. This function is orchestrated by receptor-ligand interactions that induce anti-inflammatory cytokines and repress proinflammatory cytokines [25]. Efferocytosis is triggered by multiple chemotactic factors (e.g., CXCL1, CXCL14, CCL2, CCL6–8, and CCL1) that act as ‘find me’ signals [26,27]. In addition, the accumulation of phosphatidylserine on plasma membranes marks dying cells for engulfment and degradation by macrophages [24]. The impairment of efferocytosis leads to inflammation-associated pathology and autoimmunity [28]. Although relatively little is known about efferocytosis in cancer initiation, macrophage expression of adhesion-family G protein-coupled receptor B1 (or BAI1), which recognizes phosphatidylserine ‘eat-me’ signals on cells, was recently implicated in cancer formation in a mouse model of colitis [29]. Knockout of BAI1 interfered with macrophage clearance of cellular debris, predisposed mice to colitis, and increased murine mortality. Overexpression of this gene in colon epithelial cells attenuated colitis. These observations highlighted the role of efferocytosis in resolving inflammation and presumably in attenuating colon cancer risk.

## 4. Macrophages in Parainflammation and Inflammaging 

Morbidity increases with age [30]. The process is characterized by heightened susceptibility to infectious diseases, decreased antigen-specific immunity, and a greater propensity to develop cancer. The dysregulation of innate and adaptive immune responses that occurs with aging results in low-grade inflammation [31]. This phenomenon is termed inflammaging and represents an intermediate state between overt chronic inflammation and basal homeostasis. Additional forms of subclinical inflammation have been variously referred to as parainflammation (*para* from Greek for near or adjacent) and meta-inflammation [32,33]. Meta-inflammation is associated with obesity-induced dysbiosis and intestinal barrier failure with intestinal macrophages playing a key role in metabolically induced chronic low-grade inflammation [34]. The presumption is that bacterial compounds polarize intestinal macrophages, leading to chronic low-grade inflammation, metabolic disorders, and an increased risk of cancer [33]. Parainflammation is noted in precursor lesions for CRC following a persistent DNA damage response. It was initially described in mice with a knockout of the gene for casein kinase 1 alpha 1, a negative regulator of Wnt signaling [35]. The result was activation of innate immunity with minimal recruitment of immune cells to sites of inflammation [32].

Inflammaging is a systemic process characterized by elevated levels of inflammasome-related and NF-κB-driven proinflammatory cytokines such as IL-1β, IL-6, IL-8, IL-12, C-reactive protein, and TNF-α [36,37,38]. Interestingly, anti-inflammatory cytokines such as IL-10 and TGF-β are also increased [36,39]. Inflammaging in humans correlates with poor health outcomes [40]. In pre-clinical models, inflammaging correlates with increased gut permeability, elevated levels of LPS in blood, chronic activation of TLR4, and a decreased ability of macrophages to clear bacteria and resolve infection [41,42]. The commensal microbiome is an important trigger for inflammaging. Mice housed in germ-free environments do not develop inflammaging and retain normal macrophage function [41]. Inflammaging has also been ascribed to defective efferocytosis, leading to chronic immune activation [43].

Myeloid cells are central actors in inflammaging. Macrophages readily detect subtle changes in microenvironmental tissue cues and become polarized in response [44]. As tissue microenvironments change due to aging, macrophage phenotypes also change [45]. For example, when challenged with *Candida albicans*, CD163^+^ macrophages from older adults generate lower levels of TNF-α, IL-6, and IFN-γ than do macrophages from younger individuals due to “deconditioning” of the tissue microenvironment [46]. When stimulated in vitro with ligands for TLR-1, -2, or -4, these same “aged” macrophages retain an ability to generate normal levels of TNF-α, indicating that defects in production are reversible. Conversely, macrophages from young mice can respond like “old” macrophages when exposed to senescent cells (i.e., bystander senescence) [47]. Macrophages from geriatric mice fail to induce INF-γ in T-cells, although macrophages from young or old mice do induce CD4^+^/CD8^+^ T-cell proliferation [48]. Senescent macrophages also produce elevated levels of prostaglandins by downregulating nuclear receptor RXRα [37]. Excessive prostaglandin formation contributes to T-cell suppression [37]. Finally, recruiting human and murine macrophages from bone marrow is impaired in older subjects or animals compared with younger subjects or animals [49].

Different cues in aged tissue selectively direct macrophage polarization and function. The M1 macrophage phenotype was more commonly observed in adipose and liver of 24- to 28-month-old mice than in those of younger mice [48]. In contrast, the M2 phenotype was noted in bone marrow, spleen, lymph nodes, lung, and muscle [50]. Others have noted both M1 and M2 macrophages in healthy aged tissue [48,50]. For splenic macrophages, polarization to an M1 or M2 phenotype was impaired when cells were isolated from older mice [51]. These macrophages were less responsive to pro/anti-inflammatory stimuli. Conversely, peritoneal macrophages from 14- to 15-month-old mice were more reactive to LPS and produced greater amounts of reactive oxygen species and nitric oxide [52]. These older peritoneal macrophages were more effective in eliminating infection by *Salmonella typhimurium* [52], possibly due to partial polarization. Others have conversely noted hypo-responsiveness to IFN-γ and reduced Stat-1 signaling in older macrophages [53]. In an ocular model of tissue injury, macrophages from older mice had upregulated IL-10, downregulated Fas ligand, IL-12, and TNF-α, and a proangiogenic phenotype [54]. Similarly, microglial from the brains of older mice are less responsive to TLR stimulation and constitutively secrete greater amounts of IL-6 and TNF-α than do microglia from younger mice [55]. Finally, microbiota from aged mice were shown to induce TNF-α and promote macrophage dysfunction (e.g., reducing their capacity to kill *Streptococcus pneumoniae* and increasing the production of IL-6) [41]. 

In summary, varying degrees of macrophage dysfunction have been reported with aging. Although macrophages maintain their plasticity in pre-clinical models, microenvironmental tissue cues in aging affect the correctness of polarization. The result is likely a loss of the normally healthy balance between pro- and anti-inflammatory responses. Inflammaging emerges from this milieu and increases the risk for cellular-driven cancer initiation. However, not all studies have been consistent in their observations, with differences most likely due to variation in genetic backgrounds for animals in pre-clinical models and the failure to appropriately control for host-microbiome interactions in human studies.

## 5. Macrophages in Cancer Initiation with an Emphasis on Colorectal Cancer

The transformation of somatic cells into cancer-initiating cells involves DNA damage, mutation, phenotypic changes, and proliferation. The processes are regulated by altered cell signaling and/or epigenetic changes. Carcinogens are a diverse set of agents that cause DNA damage, mutation, and abnormal cellular proliferation. In 2016, Smith et al. summarized 10 key traits for human carcinogens [56]:

(1) electrophilic reactivity;
(2) genotoxicity;
(3) genomic instability or altered DNA repair;
(4) epigenetic effects;
(5) induction of oxidative stress;
(6) induction of chronic inflammation;
(7) immunosuppression;
(8) receptor-mediated effects;
(9) transformation or immortalization; and
(10) altered cell proliferation, cell death, or nutrient supply.

Many of these characteristics are also cancer hallmarks [1]. A recent analysis of Group 1 human carcinogens that are listed by the International Agency for Research on Cancer (IARC) found that many induced genotoxicity [57]. On average, human carcinogens exhibit four or more key traits as described by Smith et al. [56]. Applying this framework to polarized macrophages suggests that they should be considered potential endogenous carcinogens. Below, we provide pre-clinical and clinical evidence that supports this concept, especially in the context of chronic inflammation, parainflammation, and inflammaging.

### 5.1. Polarized Macrophages Are Genotoxic

Multiple immune cells participate in carcinogenesis although no cell or cell function has yet been classified as carcinogenic. Immune cells that cause genotoxicity could be considered potential carcinogens. Polarized macrophages in particular have been shown to induce DNA damage and genomic instability in neighboring cells [58,59,60]. This phenomenon has been most convincingly demonstrated using irradiated macrophages. These cells produce diffusible factors that induce stress signaling, DNA damage, and chromosomal instability in neighboring cells [61]. These results have been collectively termed the radiation-induced bystander effect and observed in both animal models and humans [62,63,64].

Macrophages that are polarized by bacteria, antigens, or chemokines to an M1 phenotype can similarly induce double-strand DNA breaks [65], disrupt mitotic spindles [66], induce genomic instability [67], and/or alter DNA methylation in bystander cells (unpublished observations). Genomic damage and mutations that are induced by polarized macrophages can be blunted by lipid radical scavengers and inhibitors of COX-2. In vitro, the repetitive exposure of epithelial cells to polarized macrophages produces cancer (or tumor) stem cells and causes malignant tumors in immune-deficient mice [68]. These macrophage-derived cancer stem cells express stem-like markers (e.g., leucine-rich repeat-containing G-protein 5 [LGR5], doublecortin-like kinase 1 [DCLK1], epithelial cell adhesion molecule [EpCAM], CD44, and CD133) and markers for dedifferentiation and pluripotency [68,69].

### 5.2. Macrophage Genotoxins

Signaling proteins and other molecules generated by polarized macrophages can alter epithelial phenotype by mutating DNA, causing lipid peroxidation to generate mutagenic byproducts, and/or altering gene expression. These products include cytokines, chemokines, and growth factors important to immune defense and tissue regeneration. One example is TNF-α, a potent cytokine released by polarized macrophages that causes mutations, gene amplification, micronuclei formation, and genomic instability in epithelial cells through oxidative stress mechanisms [70]. Polarized macrophages also generate reactive oxygen and nitrogen species that have been implicated in cancer initiation [5,71,72]. Examples include superoxide, hydrogen peroxide, peroxynitrite, and hydroxyl radical, all of which can damage and mutate DNA in target cells. These reactive species are generated in part through a respiratory burst that occurs after polarization [72,73]. The carcinogenic potential of endogenous reactive oxygen and nitrogen species has been shown in mice by forming intestinal tumors without the administration of an exogenous carcinogen [74]. 

Inflammation-induced lipid peroxidation can generate diffusible breakdown products that act as stress signals and potent genotoxins [75]. 4-hydroxy-2-nonenal (4-HNE) is the most abundant reactive peroxidation product of ω-6 polyunsaturated acids [76]. This aldehyde and related lipid breakdown products are electrophilic toward cysteine, histidine, lysine, and deoxyguanosine. Adducts of 4-HNE are found in human and rodent tissues [77]. M1 macrophages generate substantial quantities of 4-HNE [66]. COX-2 also generates 4-HNE and may be a source of heptanone-etheno-DNA adducts in target cells [78,79]. In one study, inhibiting COX-2 in polarized macrophages decreased 4-HNE production [66]. Conversely, depleting intracellular glutathione—a 4-HNE scavenger—increased 4-HNE production. 4-HNE produced by polarized macrophages generates double-strand DNA breaks in vitro and is a mitotic spindle poison [66]. Finally, repetitive exposure of epithelial cells to sublethal doses of 4-HNE in vitro led to the development of cancer stem cells [68]. These cells grew as malignant tumors in immunodeficient mice. In sum, polarized macrophages can generate mutagenic electrophiles that can serve as cellular-derived carcinogens.

### 5.3. Polarized Macrophages as Drivers of Cancer Initiation

Multiple pre-clinical models have shown that polarized macrophages can express many key characteristics of carcinogens (*viz*., genotoxicity, genomic instability, epigenetic effects, immunosuppression, and cellular transformation). For example, the depletion of CX3CR1^+^ macrophages in *Apc*^Min/+^ mice that were colonized with enterotoxigenic *Bacteroides fragilis* led to a substantial reduction in colon tumor multiplicity [80]. This effect was due to the suppression of regulatory T cells and inhibition of IL-17 production. In an IL-10 knockout model of microbial-triggered colorectal carcinogenesis, the depletion of colonic macrophages by clodronate (a non-nitrogenous bisphosphonate) protected mice against inflammation and cancer [81]. This directly implicated chronically polarized M1 macrophages in cancer initiation. In a murine model with an engineered block in Stat3 signaling in macrophages, gut microflora led to robust colitis, activation of mammalian target of rapamycin (mTOR), and the development of colon cancer [82]. The activation of mTOR-Stat3 pathways in epithelial cells suggested a role for macrophages in paligenosis [83]. Another study of αν integrins on myeloid cells showed the importance of macrophages in triggering colitis and initiating colon cancer [84]. These integrins are cell-surface receptors that mediate numerous homeostatic immune responses. Mice lacking these receptors on myeloid cells had fewer regulatory T cells, increased cytokine production, severe colitis, and cancer. In other models, depleting colon macrophages or inhibiting the migration of macrophages into tissues effectively blocked intestinal inflammation and reduced/abolished tumor formation [81,85,86,87]. In addition, in many of these models, the gut microbiota polarizes macrophages that then generate reactive oxygen species and a cytokine- and radical-enriched tissue milieu. This process may contribute to cancer initiation through bystander effects [81,88]. 

The mechanisms for several human carcinogens involve polarized macrophages [89]. For example, asbestos, crystalline silica dust, and wood dust are associated with chronic inflammation. These compounds polarize macrophages to release cytokines, chemokines, and reactive oxygen and nitrogen species. These responses cause tissue injury, genotoxicity, and epigenetic alterations that lead to cancer initiation. Aflatoxin B_1_ is another human carcinogen produced by *Aspergillus* spp. that causes hepatocellular carcinoma, a major form of liver cancer. This toxin is metabolically activated to form a genotoxic epoxide that generates DNA adducts, mutates *TP53*, and induces apoptotic cell death. Debris from cell death induces COX-2 in macrophages and results in an “eicosanoid and cytokine storm” that enhances cancer cell growth [90]. Blocking both COX-2 and the soluble epoxide hydrolase suppresses cancer growth.

### 5.4. Zebrafish Models in Cancer Initiation

Zebrafish are a useful model for studying the function of myeloid cells in cancer initiation [91]. At least 70% of zebrafish genes have a human orthologue. These genes include nearly all genes known to drive human cancer. Zebrafish also have a fully functional innate immune system, can be genetically manipulated, and allow direct visualization of cell migration due to translucency during the larval stage. Myeloid-derived cells in zebrafish are developmentally similar to those in mammals and show functional conservation. These features have encouraged the use of zebrafish models in the investigation of cancer-initiating mechanisms. In general, findings show that macrophages and neutrophils promote pre-neoplastic cells at the earliest stages of carcinogenesis. The expression of just a single oncogene in zebrafish (e.g., *Akt*, *Kras*, or *Myc*) results in the rapid recruitment of macrophages and/or neutrophils to tissues and occurs prior to any clonal expansion of pre-neoplastic cells. The ingress of myeloid-derived cells has been observed in skin, liver, and brain, depending on the oncogene that is activated. Oncogene-driven carcinogenesis increases macrophage recruitment and promotes the growth of transformed cells through M2-like polarization [92,93]. Proinflammatory markers are upregulated with IL-1β and TNF-α commonly induced [91]. Finally, PGE_2_ (and presumably 4-HNE) are generated by COX-2 in these models and provide proliferative signals during mutagenesis [94]. These models consistently show macrophage recruitment to sites of cancer initiation. Similar observations have been reported in mice with Yes-associated protein strongly recruiting macrophages to tumor-initiating cells [95]. Overall, zebrafish models add unique evidence to the role of macrophages in cancer initiation and support the early recruitment of M2-like phenotypes that promote tumor development.

### 5.5. Bisphosphonates, Macrophages, and Cancer Initiation

For humans, important insights into the role of macrophages in cancer initiation have been gleaned from population studies on bisphosphonates (BPs). Non-nitrogenous BPs (e.g., clodronate, etidronate, and tiludronate) inhibit macrophages and osteoclasts by inducing apoptosis through their conversion to analogues of adenosine triphosphate [96]. In contrast, nitrogen-containing BPs (e.g., alendronate, ibandronate, and zoledronate) have multiple anti-tumor effects that include induction of tumor cell apoptosis, blocking angiogenesis, and enhancing immune surveillance [97,98]. BPs prevent fractures in patients with osteoporosis and are used as adjuncts for cancer treatment. In population studies, BPs consistently show efficacy in preventing colorectal cancer (CRC) and breast cancer [99,100,101]. In pre-clinical models of CRC, the intestinal depletion of macrophages by BPs or anti-myeloid antibodies prevents chronic inflammation and blocks carcinogenesis [81,102,103,104,105]. Presumably, these effects are the result of modulating macrophage function although the precise mechanisms remain unclear. 

A summary of key characteristics for Group 1 carcinogens that are associated with polarized macrophages is shown in Table 1. Chronic macrophage polarization as a source of endogenous carcinogenesis links these key characteristics with many of the classic hallmarks of cancer [1,56].

## 6. Macrophages as Targets for Cancer Prevention

Chronic inflammation can create a tissue microenvironment that facilitates cancer initiation [108]. There is strong evidence linking chronic inflammation to hepatic, gastric, bladder, and colorectal cancers among others [2]. Less overt inflammation, as characterized by parainflammation and inflammaging, likely also results in similar pro-carcinogenic tissue microenvironments. Accumulating evidence shows that depleting or reprogramming macrophages with probiotics or small molecule inhibitors has the potential to prevent tumor formation and limit the growth of pre-neoplastic cells. In this section, we discuss agents that may limit or prevent cancer initiation by depleting or modulating macrophages. Potential mechanisms of action include blocking recruitment, suppressing key inflammatory pathways, depleting cells, and reprogramming states of polarization. Selected representative agents for these mechanisms are listed in Table 2. Those with multiple mechanisms were classified based on considerations for the primary mechanism. Although many of the newer macrophage modulators are under clinical investigation, their long-term safety data, which is needed for cancer prevention trials, are largely unknown.

### 6.1. Agents That Block Macrophage Recruitment

Several agents have been shown to limit macrophage recruitment into sites of inflammation and reduce tumor multiplicity. These studies, however, do not necessarily establish causality for this process in cancer initiation. JNJ-40346527 is a CSF1R inhibitor that limits macrophage recruitment into the intestinal mucosa and suppresses T cell-associated colitis [109]. In tau-mediated neurodegenerative disease, this inhibitor showed anti-proliferative effects on microglia with reductions in proinflammatory cytokines [156]. Emodin is a natural anthraquinone derivative that can modulate multiple signaling pathways in macrophages. In an azoxymethane (AOM)/dextran sodium sulfate (DSS) model of inflammation-associated CRC, it reduced monocyte recruitment into inflamed tissue, reduced cytokine production, and decreased the incidence of adenomas and carcinomas [111]. Embelin is a naturally derived benzoquinone that induces apoptotic cell death through inhibition of the X-linked inhibitor of apoptosis protein [157]. In other studies, embelin and a polyclonal antibody to S100A9 protein each reduced the infiltration of macrophages into colon tissue, blocked Th17 immune responses, and reduced tumor multiplicity [112,113]. Despite such encouraging results, additional work is needed to confirm that blocking macrophage recruitment is causally linked to preventing cancer initiation.

### 6.2. Agents That Suppress Proinflammatory Pathways in Macrophages

A wide variety of small molecules and vitamins have been shown to suppress pro-inflammatory pathways in macrophages and prevent cancer initiation. In general, these agents are found to block signaling in polarized macrophages and sometimes neighboring bystander cells (e.g., NF-κB, PI3K, JNK, p38 MAP kinases, Wnt/β-catenin, and ERK1/2). This often leads to the repression of COX-2 and iNOS with reduced levels of prostaglandins, nitric oxide, TNF-α, IL-6, and IL-1β. Few of these agents, except 5-aminosalicyclic acid and vitamin D, have been tested in cancer prevention trials. 5-Aminosalicyclic acid has been used for many decades to treat ulcerative colitis. It works in part by activating AMP-activated protein kinase in macrophages and blocking JNK and p38 MAP kinase signaling [117]. These anti-inflammatory effects inhibit colon cancer initiation in murine models and help reduce the risk for CRC in ulcerative colitis [118,120]. Vitamin D is an essential human micronutrient that can modulate inflammation, proliferation, apoptosis, and immune activation. Numerous studies suggest a role of vitamin D in cancer prevention [158,159,160,161,162]. Of note, the receptor for 1,25-dihydroxyvitamin D_3_ is constitutively expressed on macrophages. Supplementation of macrophages with 1,25-dihydoxyvitamin D_3_ reduces proinflammatory cytokines [134,136]. However, a recent large clinical trial of vitamin D in older women found no decrease in all-type cancer after four years of follow-up [135]. 

### 6.3. Agents That Deplete Macrophages

BPs are commonly used in clinical practice and are known to be toxic to macrophages. These agents cause apoptosis or inhibit macrophage proliferation, adhesion, and migration [97,163,164,165,166]. Clodronate is a non-nitrogenous BP that selectively depletes macrophages via apoptosis and prevents tumor initiation in models of CRC [81,104,167]. Nitrogen-containing BPs, such as zoledronate, improve tumoricidal activity by inhibiting metalloproteases, reducing vascular endothelial growth factor binding to receptors, and modulating macrophage polarization [168]. These agents have been used to treat osteoporosis, prevent bone loss in cancer, and improve survival with cancer treatment [97,169]. Their cancer-preventive effects are observed in models of chemical- and microbial-induced cancer where inflammation and cancer formation are reduced [81,103,105,170]. In large epidemiological studies, BPs have been shown to significantly decrease the risk for CRC and breast cancer [99,100,171]. Another class of macrophage-toxic agents is represented by trabectedin, an alkaloid that binds to the minor groove of DNA and inhibits the cell cycle [137]. This compound selectively depletes monocytes, macrophages, and tumor-associated macrophages with no discernable effect on neutrophils or lymphocytes. It blocks monocytes from differentiating into macrophages and is associated with reduced angiogenesis. However, no studies have been reported that use trabectedin in cancer prevention.

### 6.4. Agents That Reprogram States of Macrophage Polarization

Reprogramming macrophage phenotypes is currently under intensive investigation in cancer therapy [172]. However, this approach has not been widely used in cancer prevention. Several small molecules are able to redirect macrophage polarization and some can block cancer initiation in murine models. These agents include resolvin D1 and licorice flavonoids. Resolvin D1 is a lipid mediator generated at sites of inflammation that bind to specific receptors on M0, M1, and M2 macrophages [145]. The result is a “proresolution” phenotype with reduced proinflammatory cytokine production and increased phagocytosis. Resolvin D1 potently suppressed cancer initiation in an AOM/DSS model and additionally acts to block cancer development as an IL-6 receptor antagonist [146]. Glycyrrhizin and licorice flavonoids suppress cancer initiation through complex effects on macrophages that involve inhibition of high-mobility group box 1 (HMGB1)-TLR4-NF-kB signaling, proinflammatory cytokines, COX-2, and M2 polarization [147,148,149]. Other agents such as BLZ945, imiquimod, and 852A can also alter macrophage polarization, but little is known of their potential in cancer prevention. 

Finally, macrophage polarization is modulated by COX-2 inhibitors. In murine models, these drugs decrease tumor multiplicity [173]. In clinical trials, they prevent colon polyps, CRC, and breast cancer [154,174,175,176]. One primary mechanism involves inhibiting the synthesis of PGE_2_ by COX-2. This leads to a suppression of M2 polarization via cyclic AMP-responsive element-binding (CREB)-mediated induction of Krupple-like factor 4 [155]. Blocking COX-2 also decreases the production of 4-HNE as a mutagenic byproduct [79]. Aspirin, an irreversible inhibitor of COX-2, decreases infiltrating of colon macrophages in the AOM/DSS model, reduces levels of IL-6 and IL-1β, and blocks iNOS expression [153]. Although COX-2 inhibitors are effective in CRC prevention, their unfavorable adverse effect profile limits their clinical utility [154]. 

### 6.5. Probiotics That Modulate Macrophage Function

**Figure 1:**
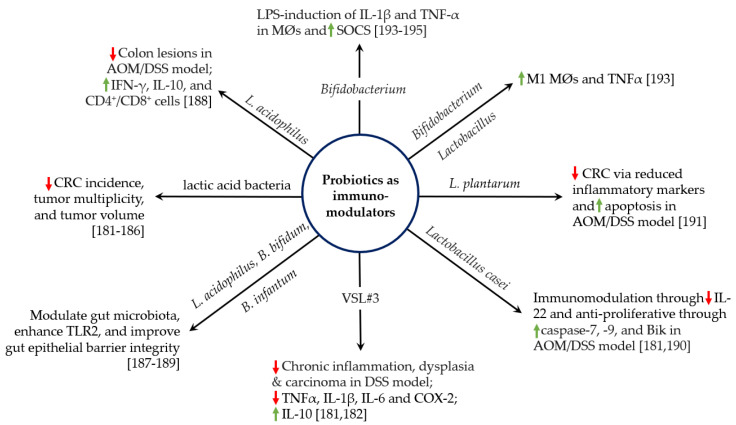
Probiotics as immunomodulators. Probiotics differently affect macrophage responses depending on their activation of varying pathogen-sensing pathways and production of metabolites. Referenced studies use a variety of well-accepted probiotics or mixtures of probiotics to regulate cellular processes such as inflammation, cytokine secretion, and macrophage polarization.

Many diseases, including cancer, are associated with a perturbation of the microbiome [177,178]. This is commonly termed dysbiosis and represents an imbalance between the healthy gut microbiota and pathobionts that promote disease. Probiotics help restore a healthy balance and have been used to prevent and treat disease [179]. Although oral probiotics work in part by out-competing pathobionts, efficacy more likely involves immune modulation, including effects on macrophage function (Figure 1) [180]. The administration of probiotic lactic acid bacteria (e.g., *Lactobacillus* and *Bifidobacterium*) have been shown to reduce CRC incidence, tumor multiplicity, and tumor volume in pre-clinical models [181,182,183,184,185,186]. Probiotics may also protect against cancer initiation by restoring epithelial barrier integrity [187]; reducing cellular proliferation, repressing COX-2, and increasing IFNγ and IL-10 [188,189]; decreasing proinflammatory cytokines (e.g., TNF-α, IL-1β, IL-22, and IL-6) [181,190]; and increasing caspase 7, caspase 9, and Bcl-2-interacting killer protein [190]. These effects can collectively produce potent cancer prevention responses. The role of immunomodulation versus gut colonization by probiotics was addressed using dead *L. plantarum* in an AOM/DSS model. Dead bacteria reduced inflammatory markers, induced apoptosis, initiated cell cycle arrest, increased IgA levels, and prevented CRC [191]. These findings suggest that oral bacterial antigens, and not necessarily colonization with live bacteria, can be sufficient for cancer prevention. However, the complex interactions among host, microbiome, and probiotics are not always so clear-cut. In an aggressive model of CRC that used AOM to initiate CRC in *Il10* knockout mice, a mixture of probiotics not only altered microbial community composition but also paradoxically enhanced tumor multiplicity [192].

The effect of probiotics on macrophage signaling and polarization has been the subject of limited investigation. In a murine model of CRC, exposing mixtures of M1 and M2 macrophages to heat-killed fractions or secreted proteins of *Bifidobacterium* and *Lactobacillus* suppressed the M2 phenotype, increased TNF-α production, and decreased tumor multiplicity [193]. In another study, macrophages exposed to *Bifidobacterium* showed greater increases in a suppressor of cytokine 1 signaling (SOCS1) and SOCS3 than macrophages exposed to LPS alone [194]. The SOCS family of proteins is an important regulator of proinflammatory cytokines in macrophages [195]. Similarly, bifidobacteria have been shown to decrease levels of LPS-induced IL-1β and TNF-α in murine macrophages. Overall, it appears that selected probiotics can reprogram macrophages toward phenotypes that block cancer initiation. However, significant gaps remain in our understanding of these mechanisms and how to best apply them to emerging strategies of cancer prevention.

## 7. Conclusions and Future Directions

Macrophages are central effectors of inflammation, parainflammation, and inflammaging, and important drivers in cancer initiation. Many cancer hallmarks and key characteristics of human carcinogens align with the polarized macrophage phenotypes. The M1 and M2 phenotypes target cells through multiple signaling pathways and generate pro-tumorigenic molecules. Evidence suggests that both phenotypes play a role in cancer initiation. Modulating these phenotypes is an emerging strategy in cancer prevention. Drugs and probiotics that target polarization, block proinflammatory pathways, or inhibit immune cell recruitment show anticarcinogenic effects. However, few new agents have been tested in clinical trials for cancer prevention. Although substantial progress has been made, additional pre-clinical modeling and clinical investigation are needed to better inform the role of macrophages in cellular carcinogenesis and cancer initiation.

## Figures and Tables

**Table 1:** Carcinogenic characteristics of polarized macrophages.

Macrophage Phenotype	Key Carcinogenic Characteristics (as Described in Ref. [56])	Cellular Targets *	Refs.
M0—tolerogenic	none	--	--
M1— proinflam-matory	electrophilic or metabolic activation	IECs	[66]
	genotoxicity	IECs, BMCs, BECs	[58,59,60,65,66,67,74]
	epigenetic alterations	IECs	(unpublished observations)
	oxidative stress	IECs	[72,74]
	chronic inflammation	IECs	[74,81,82,84,85,86]
	receptor-mediated effects	IECs	[74,85,106]
	cellular immortalization	IECs	[68,69]
	altered cellular proliferation, cell death, or nutrient supply	IECs	[82,84]
M2—anti-inflam-matory/wound healing	genotoxicity	IECs	(unpublished observations)
	immunosuppression	neuronal, skin, and liver cells in zebrafish	[80,93,94,95,107]

* IECs, intestinal epithelial cells; BMCs, bone marrow cells; BECs, bronchial epithelial cells.

**Table 2:** Selected agents targeting macrophages.

Classification of Agents	Effects on Macrophages/Effects on Other Cells	Studied in Cancer Prevention	References
**Blocking macrophage recruitment**			
JNJ-40346527 (or JNJ-527)	Blocks CSF1R and reduces recruitment of macrophages	No	[109,110]
Emodin	Reduces myeloid cell infiltration, inflammatory cytokines, and nitric oxide	Yes	[111]
Polyclonal anti-S100a9 antibody	Blocks infiltration of myeloid cells/decreases Wnt and PI3K-Akt signaling	Yes	[112]
Embelin	Inhibits X-linked inhibitor of apoptosis proteins; reduces macrophage infiltration; decreases IL-1β, IL-17a, and IL-23a/inhibits STAT3 signaling	Yes	[113]
**Suppressing proinflammatory pathways in macrophages**			
Tussilagone	Induces heme oxygenase-1; inhibits iNOS, COX-2, and TNF-α/induces apoptosis and blocks β-catenin signaling	Yes	[114,115]
BJ-3105	Activates AMP-activated protein kinase and NADPH oxidase	Yes	[116]
5-Aminosalicylic acid	Activates AMP-activated protein kinase and blocks JNK and p38 MAP kinases	Yes	[117,118,119,120]
Ursodexoycholic acid	Blocks proinflammatory signaling; reduces the production of TNF-α, IL-1β, and IL-6	Yes	[121,122,123]
Oleuropein	Suppresses COX-2 and iNOS; reduces expression of IL-1β, IL-6, TNF-α, and IL-17a/downregulates Wnt/PI3K/Akt/STAT3 signaling	Yes	[124,125]
Carvacrol	Downregulates ERK1/2 and NF-kB pathways; reduces the production of nitric oxide and expression of TNF-α and IL-1β	Yes	[126,127]
Pristimerin	Downregulates iNOS and COX-2; blocks activation of NF-κB/induces apoptosis	Yes	[128,129]
Zerumbone	Suppresses COX-2 and iNOS; blocks ERK and NF-κB; inhibits NLRP3 inflammasome	Yes	[130,131]
Pterostilbene	Suppresses COX-2, iNOS, and IL-6; blocks PI3k and NF-κB	Yes	[132,133]
Vitamin D_3_	Suppresses proinflammatory cytokines	Yes	[134,135,136]
**Depleting macrophages**			
Trabectedin	Activates caspase-8-dependent apoptosis	No	[137]
Clodronate	Forms non-functional ATP congener that promotes apoptosis	Yes	[81,104,138]
Zoledronic acid	Enhances M1 polarization; blocks farnesyl diphosphate synthase to induce apoptosis	Yes	[103,139,140]
**Reprogramming states of macrophage polarization**			
BLZ945	Blocks CSF1R and attenuates M2 polarization	No	[141]
Imiquimod	TLR7 agonist	No	[142,143]
852A	TLR7 agonist	No	[144]
Resolvin D1	Polarizes toward a pro-resolution phenotype with decreased proinflammatory cytokines and increased phagocytosis; blocks JAK2-STAT3 signaling; IL-6 receptor antagonist	Yes	[145,146]
Glycyrrhizin and licorice flavonoids	Binds high-mobility group box 1 HMGB1 to inhibit proinflammatory cytokines; blocks COX-2; blocks M2 polarization	Yes	[147,148,149]
Rosmarinic acid	Promotes M2 polarization; blocks TLR4-mediated activation of NF-κB and STAT3; suppresses the formation of reactive oxygen species and nitric oxide	Yes	[150,151,152]
Aspirin, celecoxib, and others (COX-2 inhibitors)	Inhibit M2 polarization by blocking the synthesis of PGE_2_; reduce levels of 4-HNE, IL-6, and IL-1β	Yes	[79,153,154,155]
